# Measuring Hidden Parasites in Falciparum Malaria

**DOI:** 10.1371/journal.pmed.0020268

**Published:** 2005-08-23

Approximately 40% of the world's population, mostly living in the world's poorest countries, is at risk of malaria. In the tropical and subtropical regions of the world, malaria causes 300 million acute illnesses and at least 1 million deaths annually. Ninety percent of these deaths occur in Africa, south of the Sahara, mostly among young children.

To assess disease severity, peripheral blood parasitemia is measured, but this is only a weak predictor of mortality in falciparum malaria. In addition, a microscopist is only able to count the less pathogenic circulating stages of the parasite, whereas the more pathogenic parasitized erythrocytes, sequestered in the capillaries and containing mature parasites, are not seen and therefore not counted. However, sequestered Plasmodium falciparum parasites secrete Histidine-rich protein 2 (PfHRP2), which is liberated into the plasma at schizont rupture.

In this month's *PLoS Medicine*, Arjen Dondorp and colleagues suggest that the plasma concentration of this protein might provide a better estimate for the patient's total parasite biomass and therefore be a more accurate prognostic indicator than circulating parasite load. There is evidence to support this hypothesis. A recent study by the same team measured PfHRP2 in P. falciparum cultures, and showed that approximately 89% of PfHRP2 is liberated at schizont rupture and that the variation in the amount released is limited.

In the current study the researchers measured plasma PfHRP2 concentrations in 337 patients with varying severity of falciparum malaria and, using a simple mathematical model, estimated the total body parasite biomass. This value was compared with measures of disease severity and outcome. The developmental stage distribution of circulating parasites, which also provides information on the sequestered parasites, was also evaluated in relation to plasma PfHRP2 levels in these patients.

The researchers found that the estimated geometric mean parasite burden was more than six times higher in patients with severe malaria than in patients hospitalized without signs of severe disease, and was highest in patients who died. Statistical analysis revealed that the estimated total parasite biomass was clearly associated with disease severity and outcome. By contrast, peripheral blood parasitemia and the number of circulating parasites were not associated with disease outcome, nor with other measures of severity such as admission plasma lactate concentrations.

The finding that sequestered parasite biomass is associated with disease severity fits with current thinking that sequestration of erythrocytes containing the mature forms of the parasite is the central pathological process in falciparum malaria.[Fig pmed-0020268-g001]

**Figure pmed-0020268-g001:**
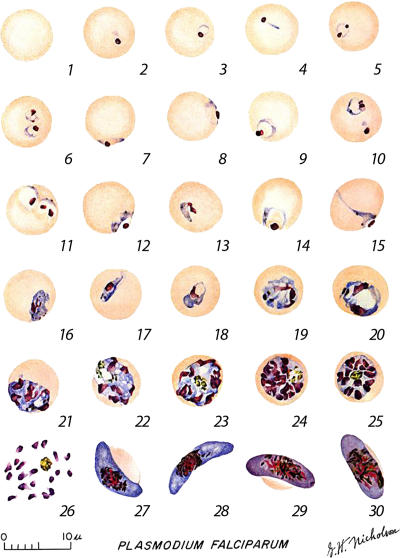
Stages in the life cycle of Plasmodium falciparum (Illustration: Coatney GR, Collins WE, Warren M, Contacos PG (1971) The primate malarias. (Bethesda): U.S. Department of Health, Education and Welfare.)

However, the team noted there were several factors that might contribute to inaccuracies in the model. For example, the amount of PfHRP2 secreted per parasite varies between different parasite strains. Also, in high transmission areas, where partial immunity against the disease develops, clearance of PfHRP2 might be increased in the presence of antibodies against the protein; in these areas—such as countries in sub-Saharan Africa—the model would thus underestimate the parasite burden and might need to be adapted further for use.

Despite these issues, estimates of plasma PfHRP2 concentrations may be useful as a research tool to stratify patients' parasite loads, say the authors. They conclude that quantitative measurements of plasma PfHRP2 in patients with falciparum malaria could be used to estimate the total parasite biomass, a parameter pivotal in the pathophysiology of the disease, and that this total parasite biomass is associated with clinical measures of the severity of the disease.

